# Supplementary material for: Are carbon emissions trading and green financial instruments synergistic? -Comprehensive quantitative research based on content analysis
Source: PLoS One. 2024 Mar 7;19(3):e0298601. doi: 10.1371/journal.pone.0298601 (PMC10919601; doi:10.1371/journal.pone.0298601)
Supplement: S1 Data — (ZIP) [file pone.0298601.s001.zip › Data/地级市Z_F工作报告.zip!/║■─╧/│ú╡┬╩╨/└┤╫╘╣1⁄2╓┌║┼í╛┬φ┐╦╩2╛▌═°í┐_2016.docx]

各位代表：

现在，我代表市人民政府，向大会报告政府工作，请连同《常德市国民经济和社会发展第十三个五年规划纲要(草案)》一并审查，请各位政协委员和其他列席人员提出意见。

“十二五”——创新创业、稳中求进的五年

“十二五”时期，是常德发展史上很不平凡、卓有成效的五年。面对错综复杂的经济形势和艰巨繁重的改革发展稳定任务，全市人民在市委的坚强领导下，扎实推进新常德新创业，总体完成了“十二五”规划主要目标任务，实现了经济社会发展稳中有进、稳中提质，为全面建成小康社会打下了坚实基础。

五年来，经济实力大为增强。地区生产总值由2010年1492亿元增加到2015年2720亿元(预计数，下同)，总量位居全省第三，年均增长11.3%。一般公共预算收入210.8亿元，社会消费品零售总额942亿元，均比2010年翻了一番。金融机构各项存款余额2220亿元，是2010年的2.3倍。五年完成固定资产投资6350亿元，是“十一五”时期的3.6倍。工业规模稳步提升。全市百亿园区达到7个、百亿产业达到8个、百亿企业达到3个，规模工业增加值突破千亿;全市高新技术企业从54家增加到97家。现代农业推进有力。“五个双百亿产业”初步形成，大宗农产品生产保持稳定，西湖西洞庭和桃源国家现代农业示范区建设扎实推进。服务业发展步伐加快。旅游、金融、商贸发展提速提质，三产业占生产总值比重达到41%，比2010年提升5.6个百分点。县域经济势头看好。桃源县、鼎城区、澧县、汉寿县、石门县地区生产总值超过200亿元，武陵区、桃源县、鼎城区、澧县、经开区、石门县一般公共预算收入超过10亿元。

五年来，城乡面貌明显改善。城镇建设迈开大步。中心城区“三改四化”成果丰硕，市本级城建投入累计超过300亿元，桃花源大桥、“三馆三中心”、白马湖公园、丁玲公园等一批重点工程相继投入使用。成功跻身全国文明城市，完美社区建设及社区网格化管理工作经验在全国全省推介。市城区建成区面积由70平方公里扩大到93平方公里，人口由70万增加到90万。各县(市)城区“三改四化”和城市创建取得明显成效。全市城镇化率达到46.9%，比2010年提高7.4个百分点。美丽乡村建设扎实推进。累计投入建设资金280多亿元，硬化农村公路2828公里，加固病险水库1043座，完成行政村电网改造1485个，实施农村土地整治173万亩，农村公共服务设施进一步完善，“3+X”农村社会治理模式创新试点稳步实施。基础设施建设全面加强。常岳、东常高速公路建成通车，中心城区80公里高速环圈完全形成，新建改造干线公路863公里，常德港盐关码头成为国家二类口岸;桃源水电站建成发电，天然气管网实现所有县(市)城区全覆盖;常德云计算中心和地理空间框架、智慧城管、智能交通、数字防控等系统建成运行，成为国家智慧城市试点城市。

五年来，改革开放不断深化。重点领域改革稳步实施。顺利完成了中央和省里部署的各项改革任务，农村土地流转体系基本建立，农村承包土地经营权抵押贷款“汉寿模式”在全国推广，文化体制综合改革工作进入全国先进行列，津市、澧县被列入国家中小城市综合改革试点和国家新型城镇化试点，桃源陬市镇纳入全国经济发达镇行政管理体制改革试点。商事制度改革、财税体制改革、医药卫生体制改革深入推进。市里自主推进的公路管理体制、园区管理体制、投融资体制、城管体制改革等取得明显成效。开放型经济加速发展。五年累计引进内资2150亿元、外资25亿美元，完成进出口总额30亿美元。积极对接央企省企和承接产业转移，新上投资过亿元项目685个;非公有制经济增加值占地区生产总值比重达到53%。对外交流合作更加活跃。新增国际友好城市2个。同时，厅市合作、校市合作、行市合作和区域合作力度进一步加大。

五年来，生态建设加速推进。节能减排得到加强。大力实施造纸、水泥、印染等行业专项整治，“十二五”节能减排任务全面完成，主要污染物排放量低于省控制指标。自然生态得到保护。启动新一轮大气污染防治行动，空气质量保持较好水平。在全省率先启动集中式饮用水源保护区划定工作，沅澧两水水质稳定在Ⅲ类以上。全国绿化模范城市创建通过国家验收，国家森林城市创建扎实推进，全市森林覆盖率达到47.98%。城乡环境得到整治。新建城镇污水处理厂11个。实施农村环境卫生整治，所有水库、大型湖泊禁止投肥养殖，农业面源污染和养殖业污染治理扎实开展。

五年来，民生福祉全面提升。城乡居民人均可支配收入分别达到24400元、11700元，是2010年的1.6倍和2.1倍。社会保障水平稳步提高。五年新增城镇就业34.2万人，新增农村劳动力转移就业40.3万人。累计支付社会保险待遇326.9亿元。城乡居民大病保险制度全面实施。城乡低保实现应保尽保，累计发放低保资金28.3亿元，分散和集中供养五保对象年人均供养标准分别达到2900元、5700元，均比2010年翻了一番。累计投入资金400多亿元，解决了34万住房困难家庭的居住问题;投入资金21.5亿元，解决了437万人的饮水安全问题，在全省率先实现城乡饮水安全全覆盖;投入资金16.1亿元，解决了31.8万人的脱贫问题。各项社会事业协调发展。投入资金30多亿元，新建改建各类学校1000多所，城乡办学条件大幅改善，湖南幼师高专、湖南应用技术学院升格挂牌，常德技师学院建设稳步推进。投入资金50多亿元，新建改建各类医疗机构3909处，市一人民医院、市一中医院等扩建项目竣工。新型农村合作医疗参合率达到98%以上，发放新农合补偿金70亿元。坚持计划生育基本国策，农村计生家庭奖励扶助、城镇独生子女父母奖励等政策顺利实施。公共文化服务体系进一步完善，在全省率先完成广播村村响工程，“三馆一站”全部免费开放，文化惠民工程深入实施，文物和非物质文化遗产保护得到加强，“百团大赛”等文体活动精彩纷呈，“扫黄打非”工作成效显著。国防动员、国防教育、人民防空和民兵预备役工作水平提高。统计、物价、粮食、民族宗教、气象、地震、档案、对台、老龄、方志、侨联、残联等各项工作取得新的成绩。工会、共青团、妇联在新常德新创业中发挥了积极作用。

五年来，民主法治持续加强。深入贯彻依法治国方略，扎实推进依法治市和“六五”普法，法治政府、法治社会、法治常德建设迈上新台阶。权力运行进一步规范。建立和执行重大决策调查研究、专家咨询、风险评估、公示听证、合法性审查等制度，实行市政府全会和常务会议邀请人大代表、政协委员、咨询专家、法律顾问列席和市民旁听制度;推行了行政执法公示制、评议制、责任制和执法过错追究制;自觉接受市人大及其常委会的法律监督和工作监督，自觉接受市政协的民主监督，主动听取各方面的意见建议，五年办理人大代表建议1132件、政协提案2181件，解决了一大批实际问题。行政效能进一步提升。大力实施“六个压减”，大力推行市直部门行政审批职能“两集中三到位”、重大投资项目联合审批等制度，大力加强社会求助服务、公共资源交易、网上政务服务系统等平台建设，大力治理涉企“三乱”、涉项“三强”等经济环境问题，查处了一批破坏发展环境的案件。政风行风进一步好转。狠抓决策执行和工作落实，严格目标管理，加强督查督办。扎实开展党的群众路线教育实践活动和“三严三实”专题教育，认真落实中央八项规定、省委九项规定和市委十项规定，加强审计监督和行政监察，查处了一批违法违纪人员。社会治理能力进一步增强。推进平安常德建设，完善社会治安防控体系，人民群众安全感持续上升。推进信访工作制度改革，落实重大决策社会稳定风险评估机制，实行领导包案化解积案，信访秩序持续平稳。严格安全生产责任制，大力开展打击非法生产和排查整治安全隐患专项行动，安全生产形势保持稳定。完善食品药品安全监管体系，实施食用油放心工程，食品药品安全保障水平提高。健全落实公共应急救援机制，处置突发事件能力得到增强。

各位代表!五年来，我们最值得珍视的是，新常德新创业的总要求内化于心、外化于行、转化于效，结出了累累硕果;我们最为感动的是，常德人民对我们共同生活的这座城市认同感越来越高，归属感越来越强，自豪感越来越足!2015——奋发有为、迎难而上的一年

2015年是实施“十二五”规划的最后一年。我们面对宏观经济下行的极大压力，积极主动适应新常态，奋发有为，迎难而上，深入推进“三大战役”，取得了经济社会发展的新突破。主要经济指标较快增长，地区生产总值增长9%，规模工业增加值增长7.2%，固定资产投资增长20%，一般公共预算收入增长14.5%，城乡居民人均可支配收入分别增长7.9%、9%。十项重大建设有效推进，石长铁路增建二线及电气化改造竣工通车;桃花源机场4D级扩建竣工投运;沅澧快速干线一号、二号、六号大道开工建设;常德烟厂易地技改项目稳步推进;常德电厂一期工程投产发电;万达广场全面开工建设;华侨城欢乐水世界盛大开园;湘雅常德医院主体工程竣工，柳叶湖现代服务业园区启动建设;建设饮水安全工程498处，解决了剩余136万人的饮水安全问题;安慈高速公路项目具备开工条件。

1、下大力引进产业项目，实体经济取得新突破。主攻重大项目建设。浪潮常德云计算中心、东星家居广场一期等项目建成运营，桃花源古镇、友阿国际广场、和瑞欢乐城等项目完成主体工程，汉能光伏、常德汽贸城、华兰德光纤等项目稳步推进，中国中车、中国兵装集团等战略投资者引进取得积极进展。全年引进内外资总额610亿元，开工建设亿元项目186个。深入推进园区攻坚。完成园区基础设施投入61亿元，新建标准化厂房150万平方米，均创历史新高。完成技改投入400亿元，新增规模工业企业101家。成功创建国家农业科技园区，国家高新区创建进入候批程序，全省创新创业园区“135”工程建设现场推进会在常德召开，推介了我市工作经验。激活消费市场需求。成功打造欢乐水世界、武陵阁步行城、沙滩公园等消费热点，高水平承办2015中国湖南国际旅游节，带旺了人气、聚集了财气、提升了名气，全年接待游客和旅游收入分别增长22.3%、26.9%。积极建设武陵移动互联网产业园，电子商务发展较快。帮扶市场主体发展。开展“千名干部联系服务千家企业”活动，与企业手挽手渡难关、闯市场。积极破解融资难题，市本级设立3亿元中小企业信贷过桥资金，帮助35家企业解决了燃眉之急;益丰大药房在上交所上市，3家企业在新三板挂牌，临澧、石门农商行挂牌营运;金融机构新增各类贷款176亿元，存贷比首次突破50%。优化用地和拆迁服务，较好地保障了重大项目用地需求。支持创新创业，新增市场主体3万户，新增家庭农场423家、农民合作社604家、规模农产品加工企业31家。

2、下大力建设海绵城市，城市提质取得新突破。从130多座城市的比拼中脱颖而出，成功入选全国首批16家海绵城市建设试点城市，3年内将获得国家专项补贴资金12亿元，海绵城市设计和79个建设试点项目已经启动。强化规划引领作用，完成了中心城区高速公路环圈内总体城市设计。加快重点项目建设，白鹤山、芦荻山和机场三大快速通道建成，柳叶湖环湖大道、桥南人防工程投入运行，穿紫河风光带、柳叶大道西延、江南风光带、沅水四桥等项目进展顺利。深入推进城市建设“三改四化”，实施项目108个，完成投入85亿元。积极推进城市管理“一改四化”，开展交通秩序、渣土运输、户外广告等专项整治，市容市貌进一步改善。持续促进城镇协调发展。各县(市)城区也按照“三改四化”“一改四化”的要求，加强城市建设，规范城市管理，城镇面貌有了很大改观。市财政安排3000万元，在鼎城灌溪镇、汉寿蒋家嘴镇、临澧新安镇、安乡黄山头镇等13个镇开展美丽城镇建设试点。完成了乡镇区划调整改革任务，以此为契机优化城镇体系，扩大城镇规模。持续推进完美社区建设。各级财政投入2.5亿元，实施建设项目218个，街道和社区公共服务硬件建设基本达标。“3+N”治理模式全面建立，社会治理信息平台、完美社区服务平台投入运行。打造社区公益服务品牌，社区服务功能得到提升。

3、下大力破解交通瓶颈，基础建设取得新突破。加快构建交通体系。黔张常高铁正在抓紧施工;常安高速复工建设;新建改建干线公路188公里，319国道桃花源景区改线、汉寿县城至军山铺、澧县城头山至张公庙等公路和安乡官垱大桥等桥梁建成通车;硬化农村公路328公里，实施危桥改造150座。强力推进水利建设。完成投入40.5亿元，创历史新高，堤防加固、中小河流治理、大型泵站改造、小型水库除险加固等项目顺利实施，小型农田水利建设深入推进;建成高标准农田15.7万亩。积极建设美丽乡村。整合各类资金3亿元，深入推进“百村示范、千村创建、村村整治”行动，100个示范村基本实现公路通组、自来水到户，涌现了石门秀坪、柳叶湖太阳谷、桃源枫树、安乡出口洲等一批示范亮点。实施蓝天碧水净土行动，全国土壤污染综合防治示范区建设稳步推进，黄石水库等水源地保护全面加强。

4、下大力增进群众福祉，民生事业取得新突破。集中力量打好民生升温战役，全面完成省重点民生工程各项指标，群众幸福指数进一步提升。全力推进精准扶贫精准脱贫。把脱贫攻坚作为重大的政治任务和民生工程来抓，广泛调动社会力量，认真落实政策措施，全市投入资金2亿元，集中办好重点贫困村“六件实事”，410个贫困村实现驻村帮扶全覆盖、干部联系帮扶贫困户全覆盖，年内脱贫12万人。深入实施教育卫生三年攻坚。教育方面，投入资金5.9亿元，改造农村薄弱学校213所，新建改建城区中小学24所，常德智慧谷加快建设，湖南幼师高专新校区投入使用。卫生方面，投入资金15亿元，建设市县医疗卫生项目55个，澧县人民医院搬迁、鼎城区人民医院扩建等项目顺利竣工，市二人民医院门急诊综合大楼、临澧县人民医院搬迁等项目完成主体工程。协调发展各项民生事业。新增城镇就业6.7万人，农村劳动力转移就业8.6万人。五大社会保险新增参保14.8万人次，开工建设保障性安居工程8.2万套，改扩建农村敬老院19所。丁玲纪念馆、桃源县文化体育中心等项目投入使用，市博物馆改扩建等项目完成主体工程。

5、下大力转换体制机制，政务服务取得新突破。以转变职能为核心推进政府机构改革。新一轮市县政府机构改革基本完成，政府部门职责交叉、权责不清等问题得到较好解决，社会管理和公共服务职能进一步强化。以简政放权为重点规范行政权力。公布政府部门权力清单、责任清单和外商投资准入管理目录、政府核准投资项目目录，市本级权力事项大幅压减。创新性地编辑出版《政策清单》，实现一册在手、政策在握，让“内行人看了更内行、外行人一看就内行”，更好地服务企业和群众。以提升效能为目标优化政务服务。清理优化审批流程，在市直12家单位开展行政审批优化流程试点，审批时限整体提速39%。加强部门内部审批职能相对集中，基本形成以窗口为主导的审批模式。规范行政审批中介服务，公布中介服务涉企收费目录。全面清理行政事业性收费项目和标准，全年为企业和群众减负2000多万元。

各位代表!

五年的发展历程充满艰辛，五年的发展成就来之不易。这得益于市委的坚强领导，得益于市人大、市政协的监督支持，得益于全市人民和社会各界的共同努力，得益于我们共同打造的最可宝贵的“常德情怀”、“常德担当”和“常德力量”。在此，我代表市人民政府，向全市人民，向各位人大代表、政协委员，向各民主党派、工商联和社会各界人士，向驻常人民解放军、武警消防官兵和公安政法干警，向所有关心支持常德发展的同志们、朋友们，表示衷心的感谢!

回顾“十二五”，我们深深体会到：无论形势怎么变化，坚持经济中心、促进常德发展，始终是我们工作的第一要务;无论困难怎么叠加，坚持改革创新、敢于争先进位，始终是我们工作的第一动力;无论任务怎么繁重，坚持民生优先、增进群众福祉，始终是我们工作的第一目标;无论考验怎么严峻，坚持风清气正、致力团结奋进，始终是我们工作的第一保障。

成绩令人鼓舞，经验弥足珍贵。在看到成绩的同时，我们也清醒地认识到，政府工作和经济社会发展仍然面临较多的困难和问题。一是工作层面有不足，在宏观经济下行的形势下，“十二五”及2015年的地区生产总值、规模工业增加值等指标没有达到预期目标。二是发展层面有短板，县域经济、非公有制经济、开放型经济、金融经济仍然是发展的薄弱环节，基础设施特别是交通设施建设欠账还较多。三是民生层面有差距，社保、住保、环保、就业、就学、就医、扶贫解困、食品安全等公共服务与人民群众的需求还不相适应。四是作风层面有顽症，一些部门衙门习气较重，办事效率低下，工作执行力不强;一些干部思想观念、能力素质还不适应新常态要求，不想为、不会为、不敢为的问题突出;“四风”问题和消极腐败现象在一些地方和单位还没有得到根治。对这些困难和问题，我们必须高度警醒，认真加以解决。“十三五”——全面小康、决战决胜的五年

今后五年，是我们深入推进新常德新创业的关键时期，是全面建成小康社会的决胜阶段。谋划和推进“十三五”发展，必须清醒认识外部环境的深刻变化，准确把握常德发展的阶段特征，主动顺应全市人民的期待和要求，科学应对发展的机遇和挑战。当前，发展环境深刻变化，国际形势错综复杂，世界经济仍然处于深度调整期，新一轮科技革命和产业变革正在兴起，我国经济发展进入速度变化、结构优化、动力转换的新常态，常德面临稳增长、促转型的双重压力、双重任务，我们必须顺应大势、积极应对，在认识适应引领新常态中把握主动权。发展机遇不断叠加，国家继续实施稳增长的政策措施，更加注重供给侧结构性改革和需求端管理，“一带一路”、长江经济带和洞庭湖生态经济区等国家战略加快实施，为我市发展带来了政策利好和重大机遇，我们必须主动对接、抢占先机，把发展的机遇转化为发展的优势。发展能量蓄势待发，全市人民围绕推进新常德新创业，瞄准建设泛湘西北现代化区域中心城市，通过强力推进“三改四化”、“三大战役”等重点工作，为常德发展蓄积了强大能量，我们必须乘势而上、奋发作为，进一步扩大发展的好势头。

“十三五”发展的指导思想是：高举中国特色社会主义伟大旗帜，全面贯彻党的十八大和十八届三中、四中、五中全会精神，贯彻省委十届十五次全会和市委六届十三次全会精神，以马克思列宁主义、毛泽东思想、邓小平理论、“三个代表”重要思想、科学发展观为指导，深入贯彻习近平总书记系列重要讲话精神，坚持“四个全面”战略布局，坚持发展第一要务，坚持创新、协调、绿色、开放、共享发展新理念，坚持新常德新创业的总要求，以提高发展质量和效益为中心，以新型工业化、信息化、农业现代化、新型城镇化、绿色化为引领，加快建成区域中心城市，提前全面建成小康社会，努力建设智慧健康美丽现代幸福新常德。

“十三五”的发展定位、发展目标、发展路径是：

1、“一个中心”的发展定位。就是从综合交通、产业集聚、商贸物流、教育医疗、科技创新、文化旅游等方面加大推进力度，加快把常德建设成为泛湘西北现代化的区域中心城市。

2、“三个迈进”的发展目标。一是建设现代交通体系，迈进“高铁时代”。告别没有高铁的历史，高速公路实现县域全覆盖，航空水运运输能力全面提升，形成现代立体交通格局。二是建成“双百城市”，迈进“大城市时代”。中心城区建成面积超100平方公里、人口超100万的大城市;津澧融城逐步实现合并设市，建成市域副中心城市。三是构建现代产业体系，迈进“千亿产业时代”。烟草业、生物科技与健康食品业、现代装备制造业产值突破千亿，现代产业体系基本形成。通过重点落实“三个迈进”的目标任务，带动实现前两年决战决胜全面建成小康社会、后三年巩固深化全面小康建设。到2020年，全市地区生产总值达到4300亿元，年均增长9%左右;规模工业增加值达到1500亿元，年均增长8.5%左右;社会消费品零售总额达到1600亿元，年均增长12%左右;一般公共预算收入达到330亿元，年均增长10%左右;固定资产投资五年累计超过1.5万亿元，年均增长17%左右。

3、“五个常德”的发展路径。一是坚持创新发展，建设智慧常德。始终把创新摆在核心位置，贯穿于经济社会发展的各个领域，努力实现经济发展由规模、要素驱动，向创新、效益驱动转换，推进城市智慧增长，努力建设具有强劲创新驱动能力、教育支撑能力、信息应用能力的智慧常德。五年内，全社会研发经费占生产总值比重达到2.3%以上，高新技术产业增加值年均增长15%左右，人均受教育年限达到10.5年，建成国家智慧城市示范城市。二是坚持协调发展，建设健康常德。按照经济社会持续健康发展的要求，在协调发展中拓宽发展空间，在加强薄弱领域中增强发展后劲，把握发展新特征，加大结构性改革力度，加快转变经济发展方式，努力建设产业协调、城乡一体、社会文明、法治健全的健康常德。五年内，新型工业化、农业现代化、服务业现代化协调发展，三次产业结构调整为10：44：46;城乡协调发展，全市城镇化率达到57%以上，实现城市人口超过农村人口的跨越;物质文明与精神文明协调发展，市民身体素质和文明素质进一步提升，文化强市建设取得更大成效。三是坚持绿色发展，建设美丽常德。坚持绿色富市、绿色惠民，深入贯彻节约资源和保护环境的基本国策，大力开展环境污染综合治理，加大生态环境保护和自然生态系统修复力度，努力建设生态宜居、人与自然和谐发展的美丽常德。五年内，水、大气、土壤等环境质量明显改善，全市空气质量优良天数达到80%以上，森林覆盖率稳定在48%。四是坚持开放发展，建设现代常德。以现代交通汇聚发展要素，以改革手段释放发展红利，以开放合作拓展发展空间，通过打通开放通道、优化开放环境，努力建设物流畅通、开放文明、充满活力的现代常德。五年内，现代立体交通体系和区域综合交通枢纽城市初步形成，以经济体制改革为引领的重点领域和关键环节改革取得突破，大开放的平台初步建成，经济发展环境进一步优化。五是坚持共享发展，建设幸福常德。牢固树立以人民为中心的发展思想，以全面建成小康社会为总揽，以社保、住保、环保和就业、就学、就医“三保三就”为重点，着力解决人民群众最关心、最直接、最现实的利益问题，使发展成果更多更公平地惠及全体人民，让人民有更多获得感，努力建设社会安定有序、人民安居乐业的幸福常德。五年内，覆盖城乡的基本医疗卫生制度和城乡一体化基本医疗保险制度更加健全，社会保险基本实现法定人员全覆盖，城镇登记失业率控制在4.5%以内，城乡居民人均可支配收入年均增长9%左右。

“十三五”期间，我们要举全市之力，集中办好关乎全局发展、惠及全市人民的“十件大事”。

1、打赢精准脱贫攻坚战。在2017年实现全市农村贫困人口整体脱贫，提前实现全面小康决战决胜。

2、建成三条高铁。实现长益常、黔张常、常岳九高铁建成通车，形成与长株潭、成渝、武汉三大城市群的快速通道。

3、建成沅澧快速干线。完成500公里沅澧快速干线建设，形成市域内快速通勤圈。

4、建成芙蓉王现代新城。常德烟厂易地技改项目竣工投产，烟草产业产值突破千亿。

5、建设四个国家级园区。把国家级常德经开区建成千亿园区、国家级常德农业科技园区建成环洞庭湖现代农业科技核心示范区，把常德高新区创建成国家级高新区、津澧开发区创建成国家级经开区。

6、建设泛湘西北教育医疗中心。高标准建设湘雅常德医院、职教大学城(智慧谷)等项目，打造泛湘西北教育医疗中心。

7、建成国家海绵城市。建设江河湖连通等工程，探索中国海绵城市建设常德模式。

8、建成白马湖商务中心。完成万达广场等项目建设，打造城市商务中心。

9、建设江南新城。建设过江隧道，实施阳明湖开发，实现江南城区提速提质发展。

10、建设旅游目的地。创建柳叶湖、桃花源、城头山、壶瓶山四大国家级旅游品牌，把常德打造成国内外知名的旅游目的地。2016——抢抓先机、扎实开局的一年

2016年，是“十三五”的开局之年，也是推进结构性改革的攻坚之年。稳增长、调结构、惠民生、防风险的要求很高，去产能、去库存、去杠杆、降成本、补短板的任务很重。我们必须抢抓机遇，乘势而上，主动作为，迎难而上，继续打好“三大战役”，强力推进“五个常德”建设，瞄准目标发力，奔着难题攻坚，全面完成改革发展稳定各项任务，夺取推进新常德新创业的更大胜利。今年经济社会发展的主要预期目标是：地区生产总值增长9%左右，规模工业增加值增长8.5%左右，固定资产投资增长20%左右，一般公共预算收入增长10%以上，城镇新增就业6.7万人以上，城乡居民人均可支配收入分别增长9%以上，完成省里下达的节能减排目标任务。

1、着力推进新型工业化

夯实园区平台。继续打好园区攻坚战役。加强园区基础建设，推行工业城市综合体建设模式，完成基础设施投入50亿元以上，建设标准化厂房150万平方米以上，做大做实一批创新创业园区。推动园区扩规提质，培育园区特色，做大园区总量，力争全市产值过200亿元园区达到3个以上、常德经开区技工贸总收入突破700亿元，力争国家级高新区创建成功。完善园区体制机制，巩固“一权两制一司”改革成果，增强园区发展活力。

促进产业升级。抢抓“中国制造2025”和湖南省“制造强省五年行动计划”的机遇，深入推进“1115”工程，全年新增百亿产业1个、亿元企业20家以上。积极支持传统优势产业转型提质，加快芙蓉王现代新城、鼎城中联起重、桃源辣妹子食品二期、临澧锦湖气门、澧县新鹏陶瓷二期、安乡晋煤金牛、津市新中意食品等项目建设，完成技改投入440亿元以上。大力发展战略性新兴产业，加快汉能光伏、华兰德光纤、力元新材、汉寿昊晖光伏、石门广电云数据中心等项目建设进度，加大中国中车、中国兵装集团、中国恒天集团等重大投资项目的引进力度，年内新兴产业产值增长13%以上。推进中小企业成长工程，力争年内新增规模工业企业100家以上。

突出科技创新。构建科技创新公共服务体系，支持企业加快工程技术研究中心、院士工作站等创新平台建设。加大产学研结合力度，实施一批重大科技专项。强化品牌和质量兴市战略，保护知识产权，支持企业参与国家标准制定、争创名品名牌。年内新增高新技术企业10家以上，专利申请量2100件以上，高新技术产业产值达到750亿元以上。

帮扶企业发展。大力开展降低实体经济企业成本行动，坚决落实国家、省关于涉企税费取消、停征、降标政策和降低企业社保、财务、电力、物流等成本的政策，坚决规范和降低中介服务收费，坚决杜绝向企业收取赞助费，最大程度减轻企业负担。深化“千名干部联系服务千家企业”活动，认真落实帮扶措施，积极开展政银企对接，完善信用担保制度，做实信贷过桥资金，政府采购优先使用本地产品，重点保障工业企业用地，市政府将尽快研究出台减轻企业负担的具体政策措施，尽最大努力帮助广大企业和企业家渡过发展难关。

2、着力推进农业现代化

坚持跳出农字抓农业、跳出农业抓农村的理念，大力抓好农业十大标志性工程，推进农业向规模化、高端化、智能化和服务化方向发展，促进农业增效、农民增收。

优化农业结构。做大做优主导产业，加快粮食、生猪、水产、蔬菜、林业“双百亿产业”发展，巩固提升大宗农产品在全省全国的地位。大力发展特色农业，促进柑橘、甲鱼、茶叶、油料等产业发展提质增效。优化农业产业区域布局，调整棉花产业结构。扩大烟叶生产规模，力争产量达到17万担以上。推进农业综合开发，建设高标准农田15万亩。

强化质量安全。推进农业标准化建设，按照操作有规程、过程有记录、产品有标识、市场有监管、质量可追溯的“四有一可”要求，建立从田间到餐桌的农业标准化体系。加强农产品质量监管，建立健全农产品质量安全监管、检测和认证体系。提升农产品品牌，采取企业主体、政府扶持、社会支持的方式，加大品牌创建、营销和推介力度，进一步提升市场竞争力和影响力。

创新经营主体。发展适度规模经营，全面完成农村土地确权登记颁证工作，依法推进土地经营权有序流转，加快培育专业大户、家庭农场、农民合作社、龙头企业等新型农业经营主体，积极培养新型职业农民。实施农产品加工业振兴工程，重点打造金健米业等龙头企业，力争全市农产品加工产值突破千亿。抓好西湖西洞庭和桃源国家现代农业示范区、常德国家农业科技园区建设，支持各区县(市)重点建设1个现代农业示范区。

完善服务保障。健全农业科技创新推广体系，加大优质种苗和先进实用技术推广力度。推进政府购买农业公益性服务，加强农机、植保、农资、农技等社会化专业服务。创新农村金融服务，健全政策性农业保险制度。强化基层动植物疫病防控体系建设。深化“农商对接”、“农超对接”，拓宽农产品销售渠道。落实强农惠农富农政策，加强农民负担监管。

3、着力推进服务业提质

突出发展文化旅游业。坚持把文化旅游作为战略性产业来抓，进一步做大做强美丽经济，塑造“亲亲常德”旅游形象。打造旅游精品，年内完成德国小镇、桃花源古镇等重点项目建设，实现锦江酒店“五一”开业、柳叶湖至穿紫河水上观光“十一”启航，加大柳叶湖环湖十大景观工程、桃花源核心景区改造、壶瓶山生态旅游等项目建设力度，启动城头山世界遗产申报工作。引进旅游品牌，加强与华侨城、山水盛典、同元集团、锦绣中华等品牌团队的合作，提升开发运营层次。深化旅游协作，推进湖南旅游“金三角”建设，加快旅游资源整合和市场拓展。全年接待游客增长20%以上、旅游综合收入增长25%以上。加快建设文化创意产业十大精品工程，促进文化创意与旅游产业融合发展。

加快发展现代金融业。积极发展和引进各类金融机构，完成常德农商行和4家县级农商行组建工作。积极拓宽融资渠道，做实做强市县投融资公司，推行PPP融资模式，扩大债券发行规模，支持有条件的企业上市融资，全年新增银行贷款170亿元以上、表外融资150亿元以上。积极创新金融产品和服务，更好地服务实体经济。促进保险业发展。优化金融生态环境，完善企业和个人征信系统，严厉打击各类非法金融活动。

大力发展商贸流通业。建成万达广场、友阿国际广场、和瑞欢乐城等城市综合体，完成武陵阁步行城二期、桥南商圈提质改造，在市城区新形成近百万平方米大型商业面积，初步构建泛湘西北区域商业中心。加强城乡物流网络建设，建设改造一批现代化大型专业市场、批发市场和物流园区，支持本土商贸物流企业做大做强。大力发展电子商务，促进线上线下融合发展，支持武陵移动互联网产业园争创省级示范基地。

协调发展其它服务业。稳定房地产业发展，建立购租并举的住房制度，加大棚改货币化安置力度，积极化解房地产库存。支持养老服务业加快发展，鼓励社会力量举办规模化、连锁化的医养护综合型养老机构，完善居家养老服务网络。发展研发设计、服务外包、节能环保、商务咨询等生产服务业，提速发展家政服务、信息通讯、娱乐休闲、体育健身等生活服务业。大力发展总部经济，支持国内外知名企业在常德建立总部或区域性总部。改造提升批发零售、住宿餐饮等传统服务业。全年社会消费品零售总额突破1000亿元。

4、着力推进新型城镇化

坚持“五个统筹”，树立智慧增长、紧凑城市理念，继续打好城市提质战役，让人民群众在城市生活得更方便、更舒心、更美好。

完善城市功能。坚持规划引领，注重产城融合，推进“多规合一”，抓好城市设计和专项规划编制，优化城市空间布局、功能布局和产业布局。坚持新区带动，建成柳叶大道西延线，开工建设过江隧道和常德大道西延线，推进沅水四桥、太阳大道、阳山大道、丹溪路等项目建设，加快实施市民之家、金融街区、阳明湖板块、丹洲生态城等带动性建设项目。坚持品质为重，把创造优质人居环境作为中心目标，深入推进“三改四化”，创建国家生态园林城市，建成江南风光带、老西门城市文化旅游商业街等项目，启动东部城区提质改造，抓好护城河西段改造、穿紫河综合治理、花山湿地改造等海绵城市建设项目。

提升管理水平。深化城市管理“一改四化”，进一步完善体制机制，将管理网格化落实到问题处置上，提高作业精细化标准，逐步推进清扫保洁、市政维护、园林绿化等运营市场化。大力发展公共交通，优化公交网络，规范出租车经营管理，推动公共自行车系统升级改造，加强立体式停车场和地下停车场建设。集中开展户外广告、渣土管理、农贸市场、夜市摊点、交通秩序、物业管理六大整治行动，还城市以整洁，给市民以方便。加强市民文明素质教育，巩固提升文明城市建设成果。

健全城镇体系。把县(市)城区作为新型城镇化的重要支点，实施县城提质扩容工程，推进“改镇设办”，增强县城集聚能力。以乡镇合并为契机，支持重点镇、卫星镇、特色镇、口子镇发展，继续推进13个美丽城镇建设试点。

5、着力推进基础设施建设

深入开展“项目推进年”活动，实施重点项目500个，完成投资1200亿元。

攻坚克难推进交通建设。全面启动交通建设五年大会战。加快黔张常高铁建设，力争长益常高铁开工建设，抓紧宜石常、常岳九、襄澧常和常桂南高铁项目前期工作。确保常安高速建成通车、安慈高速开工建设，做好益常高速复线、宜石张高速项目前期工作。加快沅水常德至桃源航道、澧水澧县至茅草街航道整治，完成德山港区千吨级码头等工程，启动和加快桃源陬市、安乡长岭洲、津市窑坡渡、鼎城蒿子港、汉寿岩汪湖等码头建设。加快沅澧快速干线一号、二号、六号大道建设，启动河洑至桃源段等项目建设。新建改建国省干线公路80公里，推进319、207国道市城区改线等公路和石龟山大桥、安乡长岭大桥、津市澧水二桥等桥梁建设。改造农村公路危桥140座。加大道路运输整治力度，始终保持公路治超的高压态势，实现公路治超常态化治理，提升公路管理和养护水平。

乘势而上推进水利建设。全年投入资金32亿元以上，完成沅澧两水140处一线大堤整治，实施江河湖库水系综合整治工程，推进蓄洪垸堤防加固、大中型灌区更新改造、大型泵站更新改造等项目建设;加强小型农田水利建设，完成115处“五小”水利建设项目，加快实施堰塘清淤、机埠整修、渠道疏浚等项目。建立饮水安全长效化常态化管理机制，争取黄石水库水资源综合利用工程开工建设。

持续发力推进能源建设。加快酒泉至湖南特高压直流线路、常德北500千伏输变电项目建设，继续实施城市配电网和农村电网改造。抓住“气化湖南”实施机遇，启动岳阳至安乡、石门至张家界天然气管网建设。开展国家新能源建设示范城市创建，加大风力发电、光伏发电等项目建设力度，抓好页岩气开发与综合利用。

加快步伐推进信息建设。做好国家智慧城市试点工作，依托大数据、物联网、云计算中心，加快构建信息资源共享交换平台，加速综合治税、信用信息、智慧旅游等应用系统建设运行。促进“三网”融合，加快光纤网络、4G基站建设，提升宽带速率和网络覆盖。深化信息化与工业化“两化”融合，推进“互联网+”行动计划，培育一批示范企业，加强数字园区和数字企业创建。

6、着力推进县域经济发展

增强县域发展活力。做好石门县省直管县下放经济社会管理权限试点，推进津澧国家新型城镇化试点和国家中小城市综合改革试点，抓好安乡国家循环经济试点，以农垦改革为契机支持西湖西洞庭加快发展。进一步完善县域经济发展考评体系，形成各具特色的县域经济板块，力争在全省县域经济发展中争先进位。

加大以市带县力度。加强县域城乡规划、产业布局、基础建设等方面的指导，促进企业向园区集中，人口、资金、技术向县城集中。加大市对县经济发展支持力度，重点支持沅澧快速干线、标准化厂房、美丽乡村、农田水利等基础设施和特色园区建设。支持在常金融机构网点向县域下沉，引导和鼓励银行机构加大对区县(市)发展的信贷投入。

打好脱贫攻坚战役。集中力量推进精准识贫、精准扶贫、精准脱贫，将扶贫精准到户、到项目、到资金、到产业，实施脱贫攻坚“六件实事”。构建专项扶贫、行业扶贫、社会扶贫工作格局，对贫困村实行驻村帮扶和干部结对帮扶全覆盖，鼓励支持各类企业、社会组织和个人参与扶贫。确保全年脱贫12万人以上。

夯实基层基础工作。深入推进完美社区建设，全面完成硬件建设扫尾，全面推行“3+N”治理模式，全面提升社区服务能力。完成新一轮并村工作，抓好村(居)委会换届选举，扩大美丽乡村“3+X”治理模式试点。完善乡村运转保障稳定增长机制，进一步改善基层工作生活条件。推行县以下机关公务员职务与职级并行制度，落实乡镇工作补贴，提高基层工作人员待遇。

7、着力推进全面改革开放

以更大的决心深化改革。按照中央和省里的统一部署，认真落实好国企、财税、价格、金融、养老保险等领域的改革举措。进一步深化行政管理体制改革、事业单位分类改革、商事制度改革，加快公共资源交易管理体制等改革。着力抓好市里自主推进的政府投资项目管理体制、城市建设体制、乡镇政府职能转变等改革。

以更宽的视野扩大开放。加强区域合作，主动对接国家“一带一路”、长江经济带和洞庭湖生态经济区战略，努力将常德打造成泛湘西北对外开放高地。搭建开放平台，提升盐关口岸运行效益，积极申报进口粮食指定口岸和桃花源机场一类航空口岸，申报建设保税物流中心。发展对外经贸，建设外贸综合服务平台，培育外贸龙头企业，鼓励“德字号”企业走出去，支持境外承揽工程，扩大劳务输出规模。强化招商引资，做好专业化、产业链、精准招商和网络招商，突出引进战略投资者，点对点抓好央企、省企和大企业大集团的对接，突出引老乡、回故乡、建家乡。力争引进内外资总额680亿元，实际利用外资6.8亿美元。

以更实的举措促进创业。大力推动大众创业、万众创新。实施全民创业计划，加强创业孵化基地、创业园区和众创空间建设，激发全社会创新创业热情。积极构建有利于“双创”并进的制度和政策环境，广泛吸引科研人员、高端人才、大学生来常创业。全面落实促进非公有制经济发展的政策，进一步消除民营资本进入的隐性壁垒，鼓励民间资本依法进入更多领域，激发非公有制经济的活力和创造力。

8、着力推进民生事业建设

完善就业社保体系。实施更加积极的就业政策，进一步加强就业培训和就业援助，突出抓好高校毕业生和就业困难人员就业。实施全民参保登记计划，加快推进社会保险全覆盖。实施城乡养老保险参保缴费激励，完成机关事业单位养老保险制度改革。推进医保城乡统筹，深化医保付费制度改革，完善工伤预防和康复机制。推动社会救助精准化，稳步提高最低生活保障标准。加快推进“两房两棚”建设，全年建设保障性住房和改造棚户区8.6万户以上。加快发展慈善事业。关心和支持残疾人工作。

加快建设教育强市。全面完成教育三年攻坚任务。办好学前教育，扩大普惠性学前教育资源，建设一批社区公办幼儿园和普惠性民办幼儿园。提升基础教育水平，抓好市一中提质改造、荷花中学新建等项目，做好北大附属学校、常德雅礼实验学校项目前期工作。支持高等教育发展，推进湖南文理学院向应用技术型大学深度转型，提升湖南幼师高专办学质量，确保常德技师学院搬进新校区办学，积极申办常德财经职院。发展现代职业教育，抓好民办教育、特殊教育、继续教育和老年教育。实施名校长、名教师、名学校工程，加强学校文化和师德师风建设，做好第二届翦伯赞教育突出贡献奖的评选和表彰工作。加大贫困家庭学生资助力度，推进留守儿童少年关爱行动计划。

加强卫生计生工作。全面完成卫生三年攻坚任务。建成湘雅常德医院，启动市第一中医院易地扩建。推进公立医院改革，完善投入补偿和运营监管机制。加强传染病等重大疾病和公共卫生监测预警与防控。抓好重性精神障碍患者救治工作，开展国家精神卫生综合管理试点。做好中医药工作。健全药品供应保障机制。扶持和规范社会办医，形成多元化办医格局。坚持计划生育基本国策，夯实基层基础，落实“全面两孩”生育政策，优化人口结构，提高人口素质。

协调发展各项事业。加强精神文明建设，健全城乡公共文化服务体系，支持文艺精品创作，严格文化市场监管。加强体育设施建设管理，促进全民健身运动，引进和举办国际马拉松赛等重大赛事。落实住房公积金缴存、提取、贷款政策，更好发挥住房保障作用。加快革命老区、边远地区、少数民族地区经济发展。做好统计、粮食、外事侨务、民族宗教、防震减灾、方志、档案、保密、对台、气象、驻外机构等工作。支持工会扩大工作覆盖面。发展老龄事业。保障妇女儿童权益，支持共青团工作。

维护大局安定和谐。加强社会治安综合治理，构建立体化防控体系，保持打击各类违法犯罪活动的高压态势。完善人民调解、行政调解、司法调解联动工作体系，做好社区矫正、法律服务、法律援助工作，启动“七五”普法。加快建设市县网上信访信息系统，继续推进领导下访包案和部门联合接访，依法及时就地解决群众合法合理诉求。实施最严格的安全生产责任制度，加强安全监管能力建设，抓好安全生产隐患排查治理，坚决防止重特大事故发生。健全食品药品安全监管机制，推进全程综合监管，严厉惩处违法行为，保障食品药品安全。完善突发公共事件应急管理机制，加强城市公共安全设施建设，提升防灾减灾和应急能力。创新网络社会管理，依法规范网络行为。

坚持军民融合深度发展，加强国防动员、国防教育、人民防空、民兵预备役和兵役工作，深入开展“双带双促”和“双拥”共建活动，巩固双拥模范城建设成果，维护保障部队和军人军属合法权益，促进军政军民团结。

9、着力推进生态文明建设

加大环境治理力度。加强大气污染防治，持续实施煤改气、煤改电工程，加大扬尘、油烟整治和黄标车、老旧机动车淘汰力度，提升空气质量优良率。加强饮用水源地保护和水环境综合治理，推进城市黑臭水体专项整治，加大畜禽养殖污染治理和禁止投肥养殖力度，争取黄石水库、毛里湖、珊珀湖等纳入国家良好湖泊保护规划，完成10个污水处理厂建设任务。严格耕地保护，实施土壤重金属污染源头治理，抓好土壤污染修复示范工程、石门雄黄矿土壤污染综合防治等项目。

改善城乡人居环境。推进市城区生态建设“八大工程”，广泛开展城乡造林绿化行动，成功创建国家森林城市。启动整市推进美丽乡村建设五年行动。加大生态文明示范区创建力度，打造一批国家生态文明建设示范村镇，开展低碳社区试点工作。开展生态文明宣传活动，增强全民生态文明意识。

大力实施节能减排。严格落实节能减排目标责任制，继续下大力整治工业企业污染物排放，实施重点污染行业清洁化改造，确保污染物排放持续下降。继续下大力淘汰落后产能，抓好工业、交通、公共机构等领域节能降耗工作，推动绿色低碳循环发展。抓好绿色建筑示范工程，促进住宅产业化。

10、着力推进政府自身建设

讲忠诚，优服务。始终坚持忠诚于组织、忠诚于人民、忠诚于职守，始终保持谦虚谨慎、戒骄戒躁的作风，全心全意为人民办实事、做好事、解难事。简政放权要更加彻底。进一步优化政府机构设置和工作流程，继续做好行政审批事项的承接落实和取消下放工作，进一步向基层放权、向社会放权、向市场放权，加强《政策清单》的学习和运用，进一步发挥政策威力。经济环境要更加优化。要像爱护自己的脸面一样爱护常德的发展环境，严厉整治中介服务环境，向各种形式的绑定服务、垄断收费、串标围标开刀;严厉整治工程建设环境，向各种形式的强行阻工、强揽工程、强买强卖开刀;严厉整治办事服务环境，向各种形式的索拿卡要、设租寻租、不作为缓作为乱作为开刀。对扰乱环境的人和事，发现一起、查处一起、曝光一起。服务群众要更加深入。扎实开展“一进二访”活动，探索推行一线工作法，完善县乡村三级便民服务网络，将政务服务不断向基层延伸，把为群众办实事、办好事变成自觉行为和工作常态。

讲创新，求突破。面对当前发展的种种困难，我们必须有新的思路。要打破思维定势，善于谋大、谋远、谋深，敢闯敢干、先行先试，在引进战略投资者、加快传统产业升级、培育新的增长点上寻求突破。面对深化改革的繁重任务，我们必须有新的办法。坚持用改革的理念、创新的举措、市场的手段，在打破利益格局、扫清体制障碍、激发内生活力上寻求突破。面对区域竞争的巨大压力，我们必须有新的作为。以舍我其谁的责任感和时不我待的紧迫感，积极扭转“被边缘化”的趋势，在化解交通瓶颈、要素制约、民生短板上寻求突破。

讲担当，重落实。真正以战略思维、宽阔视野、大局情怀、历史担当谋划和推进常德发展。真抓才能攻坚克难，实干才能梦想成真。敢于直面矛盾。蓝图一旦绘就，路子一旦选准，就要放开手脚干、顶着压力干，不回避矛盾、不掩盖问题，主动作为、积极有为。敢于强力推行。只有干出来的精彩，没有等出来的辉煌。要把抓推行落实作为一切工作的生命线，对市委市政府制定的政策、部署的任务，必须一抓到底，做到事有专管之人、时有限定之期。敢于承担责任。各级政府及其工作人员要有为了常德发展不计个人得失的胸怀，勇于负重、实干克难，不怕承担责任，不怕承担风险，推动工作毫不手软，促进发展毫不懈怠。

讲法治，强规范。政府的一切工作都要在法律框架内运行，这是一条红线，也是一条底线。严格依法行政。牢固树立宪法法律至上的理念，加快建设法治政府，严格落实重大决策程序规定，深化行政执法体制改革，抓好政府立法工作，推行法律顾问制度，建立依法行政考核评价机制。主动接受监督，自觉接受市人大及其常委会的法律监督和工作监督，执行市人大及其常委会的决议决定，认真研究处理各项审议意见，落实好规范性文件备案审查和“三统一”制度，支持和保障市政协履行政治协商、民主监督、参政议政职能，认真办理人大代表建议和政协提案，完善社情民意办理反馈机制，虚心听取和采纳各民主党派、工商联、无党派人士的意见建议，切实推动各类实际问题的解决。强化内部监察和审计监督。加强政务公开。推进重大决策部署、重要民生事项、重大投资项目和公共资源交易等领域的信息公开，提高工作透明度，打造阳光政府。

讲廉洁，守底线。只有先改变自己的态度，才能改变人生的高度。认真落实党风廉政建设主体责任，努力建设廉洁政府。坚持干净用权。对权力集中的部门和岗位，实行分事行权、分岗设权、分级授权、定期轮岗，把权力关进制度的笼子里。坚持干净干事。把纪律和规矩挺在前面，筑牢廉政防线，严格执行公务接待、办公用房等管理标准，改革公务用车制度，确保“三公”经费只减不增。坚持干净做人。深入开展反腐倡廉教育，树立高尚的政德政风，不为物欲所惑、不为人情所扰，以清正廉明的公仆形象取信于民。

各位代表!

宏伟的蓝图已经绘就，奋进的序幕已经拉开。让我们在市委的坚强领导下，把常德人民对美好生活的向往作为我们的奋斗目标，加快推进新常德新创业，为提前全面建成小康社会、谱写中国梦的常德篇章而努力奋斗!
